# β‐Cyclodextrin Inclusion Complexes with Model Pentapeptides: Role of the Tyrosine Position within the Peptide Chain

**DOI:** 10.1002/open.202500223

**Published:** 2025-06-05

**Authors:** Martina Dragone, Gianluca D’Abrosca, Antonia D’Aniello, Domenico Alberga, Getasew Shitaye, Rinaldo Grazioso, Stefano Tomassi, Luigi Russo, Roberto Fattorusso, Salvatore Di Maro, Giuseppe F. Mangiatordi, Michele Saviano, Gaetano Malgieri, Carla Isernia, Rosa Iacovino

**Affiliations:** ^1^ Department of Environmental, Biological and Pharmaceutical Sciences and Technologies University of Campania “Luigi Vanvitelli” Via Antonio Vivaldi 43 81100 Caserta Italy; ^2^ Department Human Science Link Campus Via del Casale di S. Pio V, 44 00165 Roma Italy; ^3^ National Research Council Institute of Crystallography Via Vivaldi 43 81100 Caserta Italy; ^4^ National Research Council Institute of Crystallography Via Giovanni Amendola, 122/O 70126 Bari Italy; ^5^ Department of Medicine and Health Sciences Bahir Dar University GCG2+P9X Bahir Dar Etiopia; ^6^ Department of Life Sciences, Health and Health Professions Link Campus Via del Casale di S. Pio V, 44 00165 Roma Italy

**Keywords:** β‐Cyclodextrin/peptides inclusion complexes, aromatic amino acids, binding constants, molecular docking and dynamics, spectroscopic techniques

## Abstract

Peptide's applications frequently present problems of solubility, stability, activity, or membrane permeability. To overcome these issues, cyclodextrins (CDs) can be used to form inclusion complexes with peptide hydrophobic parts; alkyl‐chains or aromatic‐rings inclusion strongly influences the interacting peptide properties. The study of model tripeptides has revealed that, among the three aromatic amino acids, tyrosine is the best suited to be included within CDs. The interaction with β‐CD of five model peptides (Tyr1‐5), each constituted by one tyrosine and four alanines, is reported: the tyrosine occupies one of the five position within each peptide chain. Among natural CDs, β‐CD has been chosen as it is the most economic, used, and only moderately toxic; its cavity size is the best suited to accommodate the tyrosine ring. Stoichiometry and affinity of each complex are evaluated and *in silico* and experimental data to describe the molecular determinants of each interaction are combined. The data further defines the role of the aromatic ring position in dictating the stability of formed complexes and demonstrates Tyr3, with its central Tyr, as the most stable complex. Noteworthy, the interaction with β‐CD induces Tyr3 to assume a U‐shaped conformation representing a nice example of conformation stabilization upon formation of inclusion complexes.

## Graphical abstract

Here, a systematic investigation of the interaction between β‐Cyclodextrin and five model peptides , each constituted by four alanines and one tyrosine differently positioned along the peptide chain, is reported. This integrated approach combines experimental and *in‐silico* techniques to describe the molecular determinants of interactions and shows how the tyrosine position within peptide sequence affects the binding affinity and complex stability.

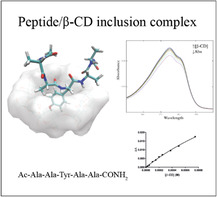

## 1 Introduction

Low molecular weights, well‐defined structures, and ability to penetrate biological barriers allow small molecules to be profitably exploited for pharmaceutical applications.^[^ ^1^ ^]^ However, in many cases the small size does not allow small molecules to target protein–protein interactions,^[^ ^2^ ^]^ which usually involve large protein surfaces.^[^ ^3^ ^]^ Peptides, on the other hand, with their larger size, modularity, and flexible backbones appear to be a better solution in these instances.^[^ ^4^ ^]^ They have a unique role in the development of new drugs thanks to their safety and tolerability in humans and represent a powerful diagnostic and therapeutic tool.^[^ ^5^ ^]^ Whether produced by means of synthetic techniques or insulated from natural sources as in the case of antimicrobial peptides, their use can be impaired by solubility, instability, or membrane impermeability issues.^[^ ^6^ ^]^ To overcome these issues there are several approaches such as their encapsulation in carbon nanotubes^[^ ^7^ ^]^ for sustained and targeted drug release, and peptide binding affinity to nanotube increases with the increase of aromatic residue content.^[^ ^8^ ^]^ Furthermore, also cyclodextrines (CDs) inclusion complexes have been proved to be a precious instrument.^[^ ^9^, ^10^ ^]^

CDs are oligosaccharides constituted by different numbers of glucopyranose units linked by α–(1 → 4) glycosidic bonds to form a cyclic assembly. The resulting structure has the shape of a truncated cone with a central hydrophobic cavity and an exterior hydrophilic surface: the wider rim is decorated with the secondary hydroxyl groups of each glucopyranose unit (C_2_ and C_3_), the narrower rim with the primary hydroxyls (C_6_).^[^ ^11^ ^]^ The cavity confers to these molecules the ability to form host/guest inclusion complexes with a large variety of compounds.^[^ ^12^ ^]^ The affinity of each guest for the given host CD will depend upon its size, geometry, and hydrophobicity as well as on the diameter of the CD cavity. This latter depends upon the number of glucopyranose units constituting the molecule. Between natural CDs, this number can vary from six in α ‐CDs to eight in γ‐CDs; β‐CD, constituted by seven units, is the most economic and commonly used and only moderately toxic.^[^ ^13^ ^]^ β‐CD can host the hydrophobic parts of a peptide and form inclusion complexes with the alkylic or aromatic side chains of the constituting amino acids, thus influencing the peptide's overall conformation, solubility, and stability.^[^ ^14^ ^]^ This interaction can in turn influence also the peptide membrane permeability and whole biological activity.^[^ ^15^ ^]^

Previous studies, performed either on insulated amino acids^[^ ^16^ ^]^ or on peptides of different sizes,^[^ ^17^ ^]^ have demonstrated that the three aromatic amino acids are the best suited to form inclusion complexes with β‐CD. Each aromatic amino acid shows a different behavior in the formation of the host/guest complexes due to its different chemical structure and steric hindrance that influence the positioning of the side chain within the CD hydrophobic cavity. Tyrosine appears to be the best suited to fit the cavity of β‐CD.^[^ ^18^ ^]^

Interestingly, apart from the characteristic of each sidechain, we have previously demonstrated that also the position of the aromatic residues within the sequence of model tripeptides has a strong influence on the formation of the inclusion complexes modulating the β‐CD/peptide binding constants (K_b_s).

Indeed, the residues flanking the aromatic residues interact with the CD outer portion and influence the positioning of the ring within the CD cavity.

For these reasons, in the present study we report by means of spectroscopic techniques, such as UV–vis and nuclear magnetic resonance (NMR) and of in‐silico molecular docking and molecular dynamics (MD) simulations, the study of the interaction between β‐CD and the five model pentapeptides reported in **Table** **1**. Each peptide consists of four L‐Alanine residues and one L‐Tyrosine differently positioned within the peptide sequence. The five peptides have been synthesized and complexed with β‐CD in aqueous solution; the stoichiometry and the affinity of each interaction has been evaluated and the structural model of each inclusion complex described. Our data allow to understand how the increase in the peptide length and the tyrosine position within the peptide chain influence the interaction with the β‐CD that in turn influences the dynamic and conformational characteristics of the peptide.

**Table 1:** Primary sequence of the synthesized pentapeptides and their abbreviation used in the text.

Name	Sequence
Tyr1	Ac‐**Tyr**‐Ala‐Ala‐Ala‐Ala‐CONH_2_
Tyr2	Ac‐Ala‐**Tyr**‐Ala‐Ala‐Ala‐CONH_2_
Tyr3	Ac‐Ala‐Ala‐**Tyr**‐Ala‐Ala‐CONH_2_
Tyr4	Ac‐Ala‐Ala‐Ala‐**Tyr**‐Ala‐CONH_2_
Tyr5	Ac‐Ala‐Ala‐Ala‐Ala‐**Tyr**‐CONH_2_

## 2 Experimental Section

### 2.1 Materials

All reagents and solvents were of analytical grade and used without prior purification. MilliQ water was used throughout the experiments. D_2_O was purchased from Sigma–Aldrich (St. Louis, MO, USA) and β‐CD from Wacker (Burghausen, Germany).

### 2.2 Synthesis of Peptides

Peptides (Table 1) were synthesized using ultrasound‐assisted Fmoc/tBu solid‐phase protocol (US‐SPPS).^[^ ^19^ ^]^

Briefly, the Rink amide AM resin (167 mg, 0.1 mmol, 0.64 mmol g^−1^) was used as the solid‐phase support. Generally, Fmoc deprotections were carried out treating the solid phase with a 20% piperidine solution in DMF under ultrasound irradiation in a SONOREX RK 52 H cleaning bath (2 × 1 min). Coupling reactions were performed employing 3 eq of amino acids preactivated with an equimolar amount of coupling reagents (HOBT and HBTU) and 6 eq of DIPEA as a base under ultrasound irradiation for 15 min. Once on‐resin linear peptide was completed, the Na‐Fmoc of the amino acid was removed and the free amino group was acetylated with acetic anhydride (38 mL, 4 eq) and DIPEA (140 mL, 4 eq) in DMF (4 mL) for 20 min. Once the reaction was completed, peptide was cleaved from the solid support with a solution of TFA/TIS (95:5, 3 mL) for 3 h at room temperature. The exhausted resin was filtered, and the crude peptides precipitated from the cleavage solution diluting to 12 mL with cold Et_2_O, and then centrifuged for two times (6000 rpm 3 15 min). After removing the supernatant, the resulting solid was dried for 1 h under vacuum, dissolved in H_2_O/ACN (95:5), and purified by Reverse‐Phase High Performance Liquid Chromatography (RP‐HPLC) equipped with a C18‐bound preparative RP‐HPLC column (Phenomenex Kinetex 21.2 mm 3 150 mm, 5 mm) (solvent A: H_2_O + 0.1% TFA; solvent B: ACN + 0.1% TFA; from 10 to 70% of solvent B over 10 min, flow rate: 10 mL/min). Fractions of interest were collected, evaporated from organic solvents, frozen, and then lyophilized. Peptides were analyzed by analytical HPLC (Agilent Technologies 1260 infinity) equipped with a C18‐bounded analytical RP‐HPLC column (Shimadzu, 5 μm‐C18–150 mm) using a gradient elution (10 − 90% acetonitrile in water (0.1% TFA) over 20 min; flow rate = 1.0 mL min^−1^; diode array UV detector). Molecular weights of compounds were confirmed by Electronspray ionization (ESI) mass spectrometry using an Agilent 6110 quadrupole Liquid Chromatography/Mass Spectroscopy (LC/MS) system and were reported, together with obtained purity and retention times, in Table S1, Supporting Information.

### 2.3 Ultraviolet–visible (UV–vis) Spectroscopy

A UV‐1700 Spectrometer (Shimadzu, Tokyo, Japan) was used with 1 cm matched quartz cuvettes. All UV–vis measurements were recorded at room temperature in the wavelength range 200–350 nm for all the five pentapeptides.

The method of the mole ratio titration was utilized to estimate the binding constants for the five pentapeptides under investigation.^[^ ^20^ ^]^ Fixed aliquots of various amounts of β‐CD solution were added to a 250 μM peptide solution. After each addition, the total absorbance A was

(1) A = εpep [peptide]  + εβ‐CD[β‐CD]  + εpep/β‐CD[peptide/βCD]

where [peptide], [β‐CD], and [peptide/β‐CD] are the concentrations of the species, respectively, and *ε*pep, *ε*β‐CD, and *ε*pep/β‐CD are the molar absorptivity (in mol^−1^ cm^−1^).

Since [peptide]_tot_ = [peptide] + [peptide/β‐CD], a combination of the Equation (1) with the definition of the binding constant of the 1:1 inclusion complex (Equation (2))

(2) $$K_{\text{1:1}}=\left[\text{peptide/β‐CD}]\text{/}[\text{peptide}\right]\left[\text{β‐CD}\right]$$

results in Equation (3)

(3) $$\Delta A=\frac{{\left[\text{Peptide}\right]}_{\text{tot}}\times K_{\text{1:1}}\times \text{Δ}\varepsilon_{\text{1:1}}\times [\beta -\text{CD}]}{1\;+\;K_{\text{1:1}}\times [\beta -\text{CD}]}$$

where Δ*A* is the absorbance difference of each peptide in the absence and in the presence of β‐CD and Δ*ε*_1:1_ = *ε*pep/β‐CD − *ε*pep − *ε*β‐CD. Each binding constant was obtained from the titration curve data reporting Δ*A* as a function of [β‐CD] fitted by nonlinear regression (Equation (3)) using the Graphpad Prism 8 software.

### 2.4 Nuclear Magnetic Resonance (NMR) Spectroscopy

All ^1^H‐NMR experiments were carried out at 600 MHz using a Bruker AVANCE III HD 600 spectrometer equipped with a triple‐resonance Prodigy N2 cryoprobe having a z‐axis pulse field gradient, located at Environmental, Biological and Pharmaceutical Sciences and Technologies Department of University of Campania “Luigi Vanvitelli” in Caserta (Italy). The lyophilized peptides were rehydrated in H_2_O/D_2_O 90/10 v v^−1^ and the pH of aqueous solutions was adjusted at physiological condition of 6.5. Final concentrations of 0.25 mM for all the peptide solutions were attained. Various amounts of β‐CD solution were added in fixed aliquots to a peptide solution.

NMR experiments for collecting structural constraints were performed at 298 K referenced to external TMS (*δ* = 0 ppm). Deuterium oxide (D_2_O) was purchased from Cambridge Isotope Laboratories (Andover, MA, USA). Mono (1D) and two dimensional (2D) spectra were accumulated with a spectral width of 5500 Hz. 2D experiments, Total Correlation spectroscopy (TOCSY),^[^ ^21^ ^]^ Rotatin‐frame Overhauser Enhancement Spectroscopy (ROESY),^[^ ^22^ ^]^ were recorded using the States–Haberkorn method. Water suppression was achieved by DPFGSE sequence.^[^ ^23^ ^]^ TOCSY and ROESY spectra were acquired with mixing times of 70, 250 ms, respectively. Typically, 64 transients of 2K data points were collected for each of the 256 increments; the data were zero filled to 2K in ω1 and ω2. Squared shifted sine‐bell functions were applied in both dimensions prior to Fourier transformation and baseline correction.

Data were processed by the Bruker Topspin program and analyzed using CARA software.^[^ ^24^ ^]^

### 2.5 Molecular Docking

Molecular docking studies were executed using AutoDock software version 1.5.7.^[^ ^25^ ^]^ The input files of each pentapeptide (guest) drawn by means of ChemDraw Ultra software^[^ ^26^ ^]^ and β‐CD (host) downloaded in the protein data bank (PDB code:1bBFN)^[^ ^27^ ^]^ format were uploaded as inputs into AutoDock and treated as ligand and receptor, respectively. The host molecule was retained as a fixed truncated‐cone structure while the guest structures were allowed to move freely. Structure refinement and energy minimization were also performed with AutoDock. A Lamarckian genetic algorithm and a grid‐based energy evaluation method were used to calculate grid maps of 40 Å × 40 Å × 40 Å and the host molecule was in the central part of the box. 50 docking calculations were performed for each guest structure. AutoDockTools,^[^ ^25^ ^]^ the AutoDock graphical interface, was used to generate the input files and to visualize the docking results. Docking calculation results of each guest molecule were clustered; the host–guest inclusion complex with lowest binding energy configuration and the highest percentage frequency was selected and analyzed using PyMOL^[^ ^28^ ^]^ and CHIMERA.^[^ ^29^ ^]^

### 2.6 Molecular Dynamics

Peptides/β‐CD complexes returned by the performed molecular docking  simulations were used as starting point for MD simulations performed using Desmond 7.7 package included in Schroedinger 2024‐1 suite.^[^ ^30^ ^]^ In particular, the peptides named Tyr1, Tyr3, and Tyr4 (Table 1) were simulated, each one in two different configurations, namely with (e.g.; Tyr1‐in) or without (e.g.; Tyr1‐out) the tyrosine residue included in the β‐CD pocket. Each of the six considered systems were solvated in an orthorhombic TIP3P water box using the Desmond System Builder tool ensuring a buffer of 15 Å from the box boundary. Na^+^ and Cl^−^ ions were added generating a 150 mM ionic concentration. All the prepared systems were minimized, equilibrated, and simulated using an isothermal isobaric ensemble (NPT, P = 1 atm, T = 310 K) with a Nosè–Hoover thermostat,^[^ ^31^ ^]^ with a relaxation time of 1 ps and a Martyna–Tobias–Klein barostat^[^ ^32^ ^]^ with a relaxion time of 2 ps. A nonbonded cut‐off of 9 Å and the OLPS4 force field^[^ ^33^ ^]^ were used. For each system, we performed three 250 ns‐long MD simulations as independent replicas (namely R1, R2 and R3) using a time step equal to 2 fs and storing the coordinates with a recording interval of 100 ps. In doing that, 7503 frames (2501 for each replica) were generated and analyzed for each system.

## 3 Results and Discussion

### 3.1 Binding Constants (Kb) and Stoichiometry of the Inclusion Complexes by UV–vis Spectroscopy

To determine the formation of the inclusion complexes, we exploited the variation in the guest chemical–physical properties, by following the UV–vis absorbance as a function of the peptide/β‐CD ratio. The continuous variation method or Job's method^[^ ^34^, ^35^ ^]^ postulates that the molar ratio *R* that represents the complexation stoichiometry corresponds to the maximum concentration of the complex. Thus, we have measured the absorbance for a series of samples prepared varying continuously the molar fraction of their components^[^ ^34^ ^]^ and determined the stoichiometry of each pentapeptide/β‐CD complex. Job's plots (Figure S1, Supporting Information) were obtained by plotting ΔAxR versus R, where Δ*A* is the difference between the absorbance measured at 280 nm before and after β‐CD addition and R = [pentapeptide]/[pentapeptide] + [β‐CD]. A 1:1 stoichiometry was found for all the studied complexes as showed by the curve maximum at *R* = 0.5.

An accurate estimation for the five K_b_s of the investigated inclusion complexes was obtained by monitoring the absorption intensity changes of the peptide as a function of the β‐CD concentration.

**Figure 1:**
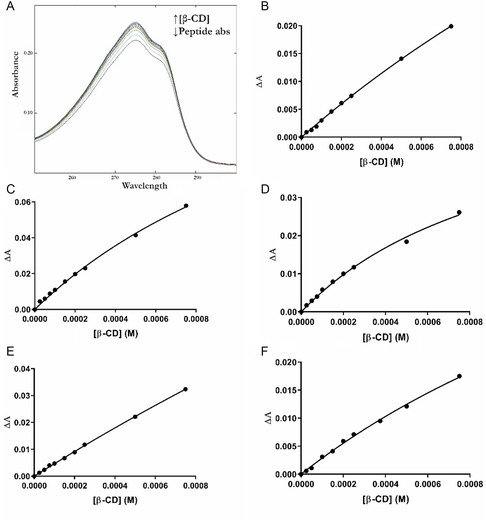
A) UV–vis spectra of Tyr3 in function of β‐CD concentration and dependence of the peptides absorbance at 280 nm from β‐CD concentration in aqueous solution at pH = 6.0 for B) Tyr1/β‐CD, C) Tyr2/β‐CD, D) Tyr3/β‐CD, E) Tyr4/β‐CD, F) Tyr5/β‐CD.

For each pentapeptide the absorbance at 280 nm was followed and a nonlinear regression estimation of the *K*_b_ used as reported in Experimental Section (**Figure** **1** and **Table** **2**).^[^ ^36^ ^]^

**Table 2:** Apparent binding constants (*K*_b_) for the pentapeptides as obtained by UV–vis spectroscopy and calculated with Equation (3).

Peptide/β‐CD	Kb(*M* ^−1^ **)**
Tyr1/β‐CD	305.3 ± 40.5
Tyr2/β‐CD	582.9 ± 87.1
Tyr3/β‐CD	944.9 ± 133.7
Tyr4/β‐CD	123.3 ± 31.7
Tyr5/β‐CD	384.2 ± 77.8

Table 2 outlines a sort of symmetrical magnitude of the Kbs which have comparable values when the aromatic chain is in position 1 or 5 (Tyr1 and Tyr5 peptides). The tyrosine affinity for the β‐CD significantly increases in Tyr3 peptide where the aromatic residue is located in position 3, in the middle of the pentapeptide sequence, while for the Tyr4 peptide a lower *K*_b_ value was obtained.

Similar *K*_b_ values were found by another study^[^ ^37^ ^]^ that complexed the β‐CD to YIGSR and YGGFL peptides and also in other previous works.^[^ ^10^ ^]^ It should be noted that the relative errors, here reported, are comparable with those shown in Caso et al. 2012, and depend on relatively weak complexation.

As expected, these data indicate that the molecular recognition of the L‐Tyr residue by β‐CD strongly depends upon the position of the side chain within the peptide sequence.

### 3.2 Nuclear Magnetic Resonance Assignments and Chemical Shift Analysis

The formation of the complexes and their characterization was, then, followed by NMR spectroscopy by measuring the proton chemical shift changes observable upon the guest–host interaction.^[^ ^38^ ^]^

The truncated cone structure of β‐CD is characterized by the hydrogens H1, H2, and H4 located on the outer surface while H3 and H5 are located inside the cavity. These latter are distinguishable because H3 is approximately closer to the wider while H5 to the narrower rim that bears also the H6 of the methylene group.

^1^H chemical shifts for each peptide were assigned both in absence and in the presence of β‐CD and in Tyr/β‐CD 1:1 and 1:3 ratios (Table S2, Supporting Information). Consistent with the estimated binding constants, all inclusion complexes are in fast‐exchange regime dynamic. As a result, their chemical shifts are a weighted average between the fractional population of free and complexed forms. This behavior allows to follow the chemical shift changes resulting from different peptide/β‐CD ratio (1:1, 1:3). Chemical shifts of all the proton atoms of the β‐CD are reported in **Table** **3**, together with the observed net variations (Δ*δ*) between the free and 1:1 complexed H3 and H5. The Δ*δ* values of both H3 and H5 protons give a strong indication of inclusion:^[^ ^39^ ^]^ when Δ*δ* for H3 is bigger than Δ*δ* recorded for H5, the aromatic ring of the tyrosine is only partially included within the β‐CD cavity; on the contrary, a Δ*δ*(H3) smaller than Δ*δ*(H5) indicates that the aromatic side chain is completely embedded. In our case, we do not observe such differences, thus indicating that in all our cases a favored aromatic amino acid position within the cyclodextrin cavity does not exist. Nevertheless, for Tyr1, Tyr3, and Tyr5 a major significant variation was observed.

**Table 3:** NMR proton chemical shifts (ppm) of the β‐CD free and complexed to each pentapeptide, at 298 K. The chemical shift variations (Δ*δ*) of the inner protons H3 and H5 with respect to the free β‐CD are indicated in brackets.

	β‐CD free	Tyr1/β‐CD 1:1	Tyr2/β‐CD 1:1	Tyr3/β‐CD 1:1	Tyr4/β‐CD 1:1	Tyr5/β‐CD 1:1	
H1	4.946	4.977	4.934	4.969	4.945	4.977	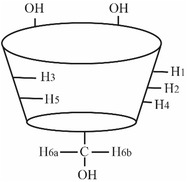
H2	3.529	3.561	3.522	3.554	3.534	3.571	
H3	3.841	3.871 (−0.03)	3.836 (0.005)	3.867 (−0.026)	3.833 (0.008)	3.873 (−0.032)	
H4	3.457	3.489	3.452	3.484	3.451	3.490	
H5	3.727	3.762 (−0.035)	3.722 (0.005)	3.755 (−0.028)	3.718 (0.009)	3.762 (−0.035)	
H6	3.753	3.784	3.752	3.776	3.747	3.785	

**Figure 2:**
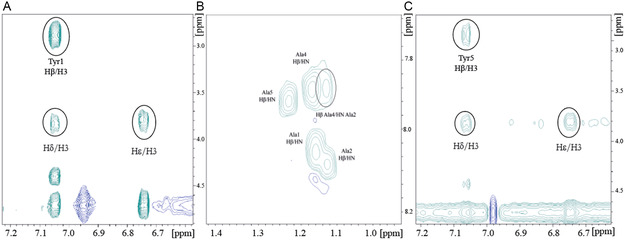
Expansion of the 2D ^1^H‐^1^H ROESY spectra of the 1:1 stoichiometry ratio complexes A) Tyr1/β‐CD, B) Tyr3/β‐CD, and C) Tyr5/β‐CD. The intra and intermolecular NOEs are labeled.

In the case of Tyr1 and Tyr5, the 2D‐ROESY spectrum (**Figure** **2**a,c) shows crosspeaks between the aromatic H*ε* and H*δ* protons of the tyrosine with the H3 hydrogen of the β‐CD indicating their proximity. In the case of Tyr3, the 2D‐ROESY spectrum does not show any detectable intermolecular connectivity with the protons of the β‐CD while intramolecular ROEs are clearly visible: Figure 2b reports the assigned cross peaks between the Ala2 NH and the Ala4 Hβ protons.

### 3.3 Molecular Docking

The structural details of the inclusion complexes under investigation have been examined by MD.

The guest peptides form a 1:1 complex with the β‐CD with comparable energies. In all the best poses (see Experimental Section), the aromatic ring is either fully or partially embedded in the β‐CD hydrophobic cavity (Figure S2, Supporting Information). As expected, the tyrosine sidechain enters the wider rim of the β‐CD cavity.

**Figure 3:**
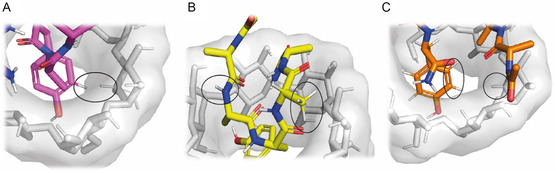
Aromatic ring close view of A) Tyr1, B) Tyr3, and C) Tyr5 complexed with β‐CD. The hydrogens of the peptide/β‐CD giving rise to ROE cross peaks are labeled.

The complex involving Tyr1, which exhibits the most populated clustering (35/50 poses) among the obtained models, shows that the tyrosine ring is fully inserted in the cavity and interacts with the β‐CD lower rim. Similarly, Tyr5 aromatic moiety deeply inserts the cavity and establishes interactions with the lower rim (Figure S2, Supporting Information). Both complexes align well with the ROE data (**Figure** 2 and **3**). Tyr2 and Tyr4, aside from minor differences in the orientations of the aromatic rings, exhibit similar behavior in the formed complexes (Figure S2, Supporting Information).

Interestingly, when the tyrosine residue is at the center of the peptide sequence (i.e., in the case of Tyr3) the aromatic sidechain fits into the cavity with an orientation of the ring parallel to the plane of both rims. Of note, in the most representative pose, upon the tyrosine inclusion, the peptide appears to be stabilized in a sort of U‐shaped conformation that brings Ala2 closer to Ala4 in clear agreement with the ROE contacts shown in Figure 3B.

These findings clearly indicate that the pentapeptides are characterized by slightly different binding modes that are likely to explain the variation of the binding affinity observed.

Although NMR data were not considered in the generation of the complexes, the good matching between the models obtained and the NMR data, both obtained in an unbiased way, validates the overall description of the molecular recognition mechanism underlying the peptides/β‐CD interaction. When the tyrosine is at the N‐ or at the C‐terminal end of the peptide, the aromatic sidechain enters the cavity, its hydroxyl group interacts with the β‐CD lower rim while the flexibility of the peptide backbone allows the remaining part of the peptide to lie on the upper rim. On the other hand, when the tyrosine is at the center of the peptide sequence, its interaction with the host molecule induces an overall structural rearrangement of the guest.

Due to Tyr1 and Tyr5 similarities in terms of estimated affinities and docking results, we have further investigated the behavior of Tyr1, along with Tyr3 and Tyr4 that respectively exhibited the highest and lowest Kb values.

### 3.4 Molecular Dynamics

The above described docked complexes formed by Tyr1, Tyr3, and Tyr4 were simulated by means of MD simulations (hereafter named Tyr1‐in, Tyr3‐in and Tyr4‐in) to analyze their stability during the time of the simulation. To support our description, simulations were also run on the lower‐energy poses with the aromatic moiety not included in the β‐CD cavity (hereafter Tyr1‐out, Tyr3‐out, and Tyr4‐out).

**Figure 4:**
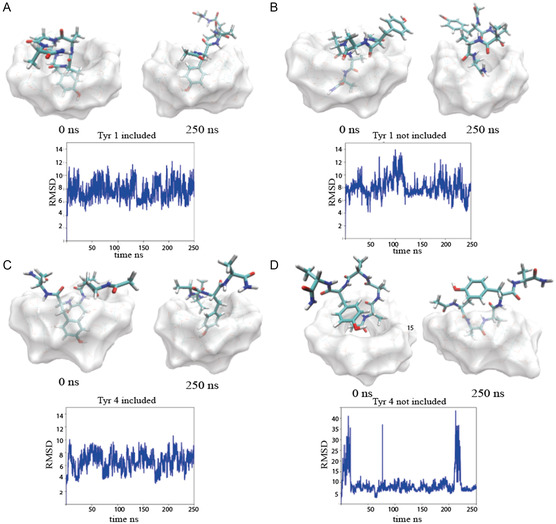
Simulated models at 0 ns and 250 ns along MD simulations trajectory with RMSD timing evolution of the complexes: A) Tyr1‐in; B) Tyr1‐out; C) Tyr4‐in; D) Tyr4‐out.

**Figure 5:**
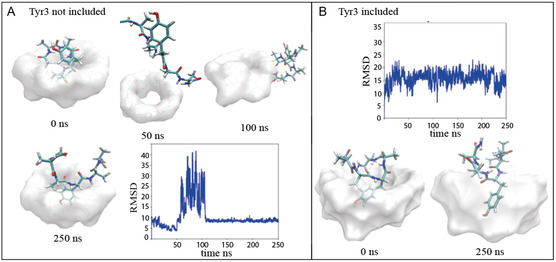
A) Simulated models of Tyr3 not included complex along the trajectory at 0 ns, 50 ns, 100 ns, and 250 ns with RMSD timing evolution. B) Simulated models of Tyr3 included complex along the trajectory at 0 ns and 250 ns with RMSD timing evolution.

The trajectories were analyzed to compare the different conformations as well as the stability of the six different complexes under investigation. The RMSD values were computed using the complexes obtained from MD simulations as the reference conformation and aligning the trajectory over the heavy atoms of β‐CD. RMSD time evolution for the peptides in the different simulated systems is shown in **Figure** **4** and **5**.

The RMSD plots, along with the models extrapolated from specific frames of the MD trajectory, clearly show that, when the tyrosine residue is not included in the β‐CD pocket, the complex is unstable. For example, the high RMSD values observed in the Tyr4‐out trajectory (see peaks in Figure 4D) indicate complex dissociation during the simulation. This instability is particularly evident in the second run of the simulation (R2), where all the systems with an initial configuration showing tyrosine outside the β‐CD pocket exhibit high RMSD values and complex disruption.

Tyr3/β‐CD complex shows a preferential conformation in the docking models and the higher *K*_b_ value. Interestingly, during MD simulation of the Tyr3‐out system (**Figure** **5**A) the complex dissociates after 50 ns, the peptide completely detaches from the surface of the β‐CD and after 105 ns reforms a new complex with the tyrosine residue included in the β‐CD pocket. This behavior underlines the high propensity of this peptide to be included in the β‐CD cavity in clear accordance with the experimental data.

Remarkably, in the simulation performed starting from the Tyr4‐out system, a Tyr4‐in conformation is reached without a total disruption of the complex but rather following a peptide conformational rearrangement only.

On the contrary, the complexes with the tyrosine included into the β‐CD (Tyr1‐in, Tyr3‐in) are stable throughout the trajectory with the exception of the Tyr4‐in system whose trajectory shows a complex destabilization at the end of the simulation. Accordingly, the Tyr4 system is also the least stable in our experimental characterization.

The higher stability of the Tyr‐in conformations is also supported by the computation of the RMSF values reported in **Table** **4**. Indeed, Tyr3‐in and Tyr4‐in return lower RMSF value with respect to the corresponding systems showing the tyrosine residue outside the β‐CD pocket (i.e.: Tyr3‐out and Tyr4‐out). Remarkably, among the different systems, Tyr3‐in is the one responsible for the lowest RMSF values (always <5.00 Å, irrespective of the considered replica), in full agreement with the experimental results.

**Table 4:** Average per residue RMSF (Å) calculated over 250 ns long MD trajectories.

System	R1	R2	R3
Tyr1‐in	6.42	6.50	6.61
Tyr3‐in	4.73	4.72	4.67
Tyr4‐in	4.66	5.74	4.61
Tyr1‐out	5.25	6.61	7.87
Tyr3‐out	10.83	5.48	5.33
Tyr4‐out	8.51	17.66	8.21

We also computed the radius of gyration over the produced trajectories for each peptide/β‐CD complex (Table S3, Supporting Information) as well as for the peptide taken alone (Table S4, Supporting Information).

Remarkably, the low values returned by Tyr3‐in can be related to a large number of interactions between the peptide and β‐CD, in turn indicative of a high binding strength. This can be quantified in **Table** **5** where the number of contacts (H‐bonds, hydrophobic, and water bridges) between β‐CD and the considered peptides, normalized with the number of analyzed frames, is reported for each of the considered systems. As expected, Tyr3‐in system returns the greatest number of interactions.

**Table 5:** Total number of contacts (H‐bonds, hydrophobic, and water bridges) between β‐CD and peptides normalized with the number of analyzed frames.

System	R1	R2	R3
Tyr1‐in	5.02	4.80	4.91
Tyr3‐in	5.45	5.43	5.29
Tyr4‐in	5.12	4.72	5.20
Tyr1‐out	4.45	4.28	4.31
Tyr3‐out	4.27	4.77	4.45
Tyr4‐out	3.95	2.20	4.50

Finally, the low radius of gyration returned by Tyr3‐in can be related to the presence of contacts between the residues Ala2 and Ala3, as found in the described NMR experiments. This is confirmed by MD simulations as we found in the analyzed trajectories the presence of at least one contact (distance less than 4 Å) between the protons of these residues in 59.47% (R1), 64.75% (R2), and 41.66% (R3) of the analyzed frames. For comparison, percentages as low as 29.70% (R1), 38.58% (R2), and 34.19% (R3) were returned by Tyr3‐out. All these data, taken together, strongly support the conformation displaying a tyrosine residue inside the β‐CD pocket as the most stable one. Furthermore, the found consistency with the experimental results strongly supports the robustness of the performed MD simulations.

## 4 Conclusion

Solubility, instability, and low membrane permeability represent major issues in the development of peptides‐based drugs.^[^ ^40^ ^]^ Thus, the quest for supramolecular binders capable to tackle these problems by interacting with specific amino acids or their sidechains represents an important step.^[^ ^41^ ^]^ CDs have been proved to be a precious instrument in this context as their hydrophobic cavity and hydrophilic surface allow them to form inclusion complexes with a large variety of compounds in aqueous solutions.^[^ ^42^ ^]^ They have been proved capable to include both specific amino acid sidechains within a peptide sequence or free amino acids not involved in peptidic bonds.^[^ ^43^ ^]^ We have previously focused our attention on the three aromatic amino acids (Tyr, Trp, and Phe) as they are not only particularly important in peptides and proteins interactions but also in analytical studies thanks to their UV–vis absorption bands and fluorescence properties.

Using model tripeptides, each constituted by two alanine and one of the three aromatic residues in different position, we have demonstrated that the behavior of each aromatic moiety is quite different in terms of interaction with the β‐CD and that, in turn, the interaction mode with this latter changes with the change of the position of the aromatic amino acid within the peptide sequence.^[^ ^17^ ^]^ The affinities measured for the three peptides containing the tyrosine residue appear to be in all the cases higher. The tyrosine side chain enters the CD through the wider rim, its –OH group guides the aromatic ring to deeply penetrate the β‐CD hydrophobic cavity, and stabilizes the peptide/β‐CD inclusion complex by interacting with the CD lower rim. Moreover, we have demonstrated that, among the three possible positions, the most stable complex is formed when the aromatic ring is in the center of the sequence. The inclusion of the hydrophobic aromatic ring in this position allows the flanking alanine residues to interact with the hydroxyl group of the β‐CD and further stabilize the formed complex.

We have here explored, by means of a theoretical and experimental approach, how the increase in the peptide length and in the number of possible positions influences the formation of the complex and of the dynamic and conformational features of the peptide with it. We have demonstrated the 1:1 stoichiometry of each formed complex by means of Job's method and evaluated the magnitude of its binding constant via UV–vis. We have also described the molecular details of each interaction in both silico, by means of molecular docking and MD, and experimentally via 2D NMR spectroscopy. Our data show how the behavior, the stability, and the Kbs of the β‐CD/peptide system change with the modification of the tyrosine position within the peptide sequence. Tyr3, the peptide bearing the aromatic residue in central position, also in this case forms the most stable complex with the highest affinity for the β‐CD host. The complex is stabilized by the interaction of the flanking residues with the CD upper rim. Important differences with respect to the previous tripeptides study are shown when the tyrosine residue is at the N‐ or at the C‐terminal (Tyr1 and Tyr5, respectively). In the tripeptides the tyrosine in the first position did not align with the central axis of the CD while the tyrosine in the last position did not penetrate the cavity at all. In the case of the pentapeptides here reported, the extended length of the host peptides allows the aromatic ring to align with the longitudinal axis of the host molecule and facilitates the deep penetration into the β‐CD hydrophobic cavity. Another interesting difference is found when the tyrosine is in central position: Tyr3 is arranged in a sort of U‐shaped form indicating that the β‐CD preferentially stabilizes this conformation.

In conclusion, our study outlines the importance of slight details of a peptide sequence in determining the formation of stable inclusion complexes. These same details should be kept in the proper consideration when designing a peptide drug or when trying to modulate a CD/peptide interaction. The propensity of the Tyr residue to be included in the β‐CD demonstrated by our data can be extrapolated for the possible design of active peptides by inserting tyrosine residues in the appropriate position.

## Conflict of Interest

The authors declare no conflict of interest.

## Supporting information

Supplementary Material

## Data Availability

The data that support the findings of this study are available in the supplementary material of this article.
