# Supplementary material for: β‐Cyclodextrin Inclusion Complexes with Model Pentapeptides: Role of the Tyrosine Position within the Peptide Chain
Source: ChemistryOpen. 2025 Jun 5;14(10):e202500223. doi: 10.1002/open.202500223 (PMC12518031; doi:10.1002/open.202500223)
Supplement: Supplementary file 1 — Supplementary Material [file OPEN-14-e202500223-s001.pdf]

Supporting Information

β-Cyclodextrin Inclusion Complexes with Model Pentapeptides: Role of the Tyrosine Position Within the Peptide Chain

Martina Dragone§ [a], Gianluca D'Abrosca§ [b], Antonia D'Aniello[c], Domenico Alberga[d], Getasew Shitaye[a] [e], Rinaldo Grazioso[a], Stefano Tomassi[f], Luigi Russo[a], Roberto Fattorusso[a], Salvatore Di Maro[a], Giuseppe F. Mangiatordi[d], Michele Saviano*[c], Gaetano Malgieri[a], Carla Isernia*[a], Rosa Iacovino*[a]

Dedicated to the memory of Prof. Luis Moroder

[a] M. Dragone, G. Shitaye, R. Grazioso, L. Russo, R. Fattorusso, S. Di Maro, G. Malgieri, C. Isernia, R. Iacovino
Department of Environmental, Biological and Pharmaceutical Sciences and Technologies
University of Campania "Luigi Vanvitelli"
Via Antonio Vivaldi 43, 81100 Caserta, Italy
E-mail: rosa.iacovino@unicampania.it, carla.isernia@unicampania.it

[b] G. D'Abrosca
Department Human Science
Link Campus
Via del Casale di S. Pio V, 44, 00165 Roma, Italy

[c] A. D'Aniello, M. Saviano
Institute of Crystallography
National Research Council
Via Vivaldi 43, 81100 Caserta, Italy
E-mail: michele.saviano@cnr.it

[d] D. Alberga, G.F. Mangiatordi
Institute of Crystallography
National Research Council
Via Giovanni Amendola, 122/O, 70126 Bari, Italy

[e] G. Shitaye
Department of Medicine and Health Sciences
Bahir Dar University
GCG2+P9X, Bahir Dar, Etiopia

[b] S. Tomassi
Department of Life Sciences, Health and Health Professions
Link Campus
Via del Casale di S. Pio V, 44, 00165 Roma, Italy

Table S1. Sequences and analytical data of the synthesized peptides.

Name	Sequence	Purity*	tR*	M.W. Calculated	M.W. Found **
**Tyr5**	Ac-Ala-Ala-Ala-Ala-Tyr-$CONH_2$	≥ 95%	10.045	506.55	507.30(+H^+) 529.15 (+Na^+)
**Tyr4**	Ac-Ala-Ala-Ala-Tyr-Ala-$CONH_2$	≥ 95%	10.012	506.55	507.30(+H^+) 529.15 (+Na^+)
**Tyr3**	Ac-Ala-Ala-Tyr-Ala-Ala-$CONH_2$	≥ 95%	11.898	506.55	507.25(+H^+) 529.25 (+Na^+)
**Tyr2**	Ac-Ala-Tyr-Ala-Ala-Ala-$CONH_2$	≥ 95%	10.288	506.55	507.30(+H^+) 529.15 (+Na^+)
**Tyr1**	Ac-Tyr-Ala-Ala-Ala-Ala-$CONH_2$	≥ 95%	10.081	506.55	507.30(+H^+) 529.15 (+Na^+)

**Peptides were analysed by analytical HPLC (Agilent Technologies 1260 infinity) equipped with a C18-bounded analytical RP-HPLC column (Shimadzu,5µm- C18-150 mm) using a gradient elution (10−90% acetonitrile in water (0.1% TFA) over 20 min.*
*** Molecular weights of compounds were confirmed by ESI-mass spectrometry using an ESI-mass spectrometry using a Shimadzu LCMS 2020 system.*

Table S2. NMR proton chemical shifts (ppm) for all the pentapeptides both in the free form and in the presence of β-CD in Tyr/β-CD 1:1 and 1:3 ratios.

Residue	HN	Hα	Hβ	Others
**Tyr1 free**	8.177	4.487	3.001-2.935	7.145-6.844
Ala2	8.162	4.266	1.396	
Ala3	8.243	4.299	1.396	
Ala4	8.268	4.280	1.322	
Ala5	8.193	4.274	1.399	
**Tyr1/β-CD 1:1**	8.172	4.494	3.004-2.931	7.136-6.835
Ala2	8.122	4.228	1.387	
Ala3	8.194	4.269	1.393	
Ala4	8.241	4.294	1.391	
Ala5	8.190	4.262	1.322	
**Tyr1/β-CD 1:3**	8.143	4.457	2.983-2.893	7.112-6.802
Ala2	8.097	4.201	1.354	
Ala3	8.210	4.260	1.355	
Ala4	8.255	4.251	1.365	
Ala5	8.167	4.234	1.361	

Residue	HN	Hα	Hβ	Others
Ala1	8.038	4.232	1.289	
**Tyr2 free**	8.050	4.510	3.030-2.920	7.070-6,769
Ala3	8.146	4.231	1.362	
Ala4	8.109	4.218	1.352	
Ala5	8.180	4.169	1.215	
Ala1	8.027	4.225	1.288	

**Tyr2/β-CD 1:1**	8.055	4.514	3.033-2.922	7.056-6.751
Ala3	8.142	4.224	1.362	
Ala4	8.107	4.212	1.352	
Ala5	8.179	4.167	1.215	
Ala1	8.004	4.214	1.283	6.802
**Tyr2/β-CD 1:3**	8.061	4.516	3.038-2.924	7.059-6.759
Ala3	8.133	4.224	1.354	
Ala4	8.106	4.109	1.349	
Ala5	8.179	4.159	1.217	

**Residue**	**HN**	**Hα**	**Hβ**	**Others**
Ala1	8.215	4.110	1.217	
Ala2	8.271	4.139	1.186	
**Tyr3 free**	8.020	4.401	2.884-2.919	7.018-6.726
Ala4	8.006	4.148	1.228	
Ala5	8.044	4.095	1.293	
Ala1	8.219	4.115	1.217	
Ala2	8.272	4.136	1.186	
**Tyr3/β-CD 1:1**	8.005	4.408	2.926-2.887	7.020-6.730
Ala4	7.994	4.152	1.227	
Ala5	8.038	4.107	1.293	
Ala1	8.207	4.112	1.226	
Ala2	8.269	4.129	1.182	
**Tyr3/β-CD 1:3**	8.008	4.406	2.938-2.886	7.020-6.720
Ala4	7.982	4.154	1.234	
Ala5	8.047	4.108	1.296	

**Residue**	**HN**	**Hα**	**Hβ**	**Others**
Ala1	8.196	4.234	1.332	
Ala2	8.138	4.234	1.270	
Ala3	8.227	4.232	1.304	
**Tyr4/free**	8.015	4.495	3.000-2.997	7.115-6.815
Ala5	8.179	4.222	1.328	
Ala1	8.193	4.233	1.331	
Ala2	8.134	4.216	1.270	
Ala3	8.215	4.229	1.305	
**Tyr4/β-CD 1:1**	8.002	4.487	2.995-2.984	7.108-6.809
Ala5	8.174	4.239	1.325	
Ala1	8.187	4.224	1.330	
Ala2	8.119	4.211	1.270	
Ala3	8.209	4.227	1.290	

**Tyr4/β-CD 1:3**	8.000	4.482	2.990-2.945	7.103-6.791
Ala5	8.162	4.220	1.324	

Residue	HN	Hα	Hβ	Others
Ala1	8.160	4.155	1.269	
Ala2	8.077	4.137	1.237	
Ala3	8.222	4.179	1.276	
Ala4	8.046	4.134	1.197	
**Tyr5 free**	7.888	4.426	2.988-2.878	6.751-7.062
Ala1	8.159	4.153	1.270	
Ala2	8.079	4.137	1.229	
Ala3	8.215	4.173	1.278	
Ala4	8.055	4.133	1.198	
**Tyr5/β-CD 1:1**	7.883	4.426	2.986-2.879	6.747-7.063
Ala1	8.160	4.149	1.273	
Ala2	8.082	4.136	1.229	
Ala3	8.217	4.181	1.278	
Ala4	8.062	4.134	1.210	
**Tyr5/β-CD 1:3**	7.898	4.425	3.000-2.882	6.745-7.062

Table S3. Radius of gyration (nm) of the investigated complexes returned by the performed 250-ns long MD simulations.

System	R1	R2	R3
Tyr1-in	6.41±0.15	6.91±0.15	6.92±0.33
Tyr3-in	6.83±0.35	6.39±0.16	6.45±0.22
Tyr4-in	6.44±0.21	6.56±0.41	6.45±0.21
Tyr1-out	6.75±0.45	6.59±0.48	6.88±1.24
Tyr3-out	7.34±2.37	6.50±0.27	6.60±0.38
Tyr4-out	7.11±1.50	9.47±3.13	6.93±1.55

Table S4. Radius of gyration (nm) of the peptides returned by the performed 250-ns long MD simulations.

System	R1	R2	R3
Tyr1-in	5.82±0.53	5.94±0.56	5.97±0.51
Tyr3-in	5.19±0.31	5.15±0.36	5.07±0.38
Tyr4-in	5.14±0.46	5.23±0.40	5.17±0.45
Tyr1-out	5.38±0.66	5.95±0.57	5.67±0.69
Tyr3-out	5.29±0.35	5.14±0.42	5.27±0.42
Tyr4-out	5.41±0.40	5.30±0.43	5.18±0.42

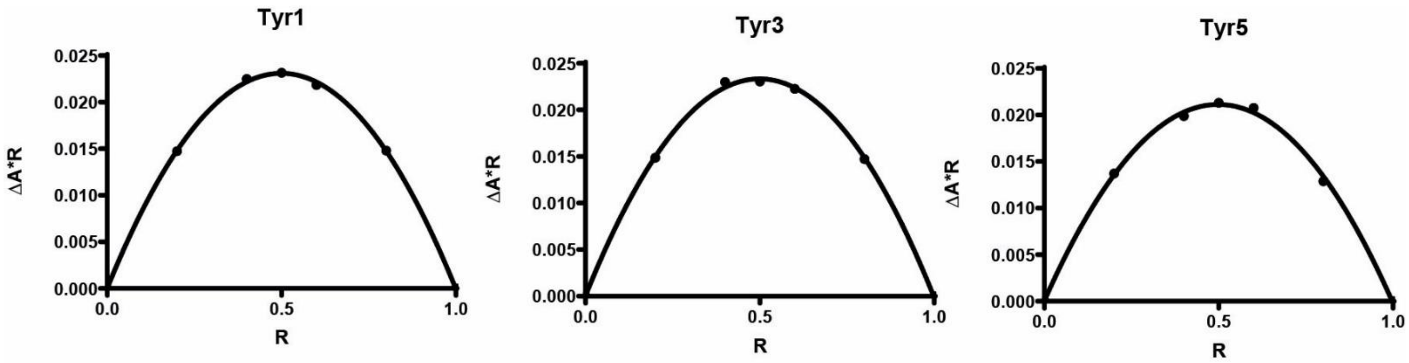

Figure S1. Job plot at □max 275 nm for Tyr1, Tyr3 and Tyr5.

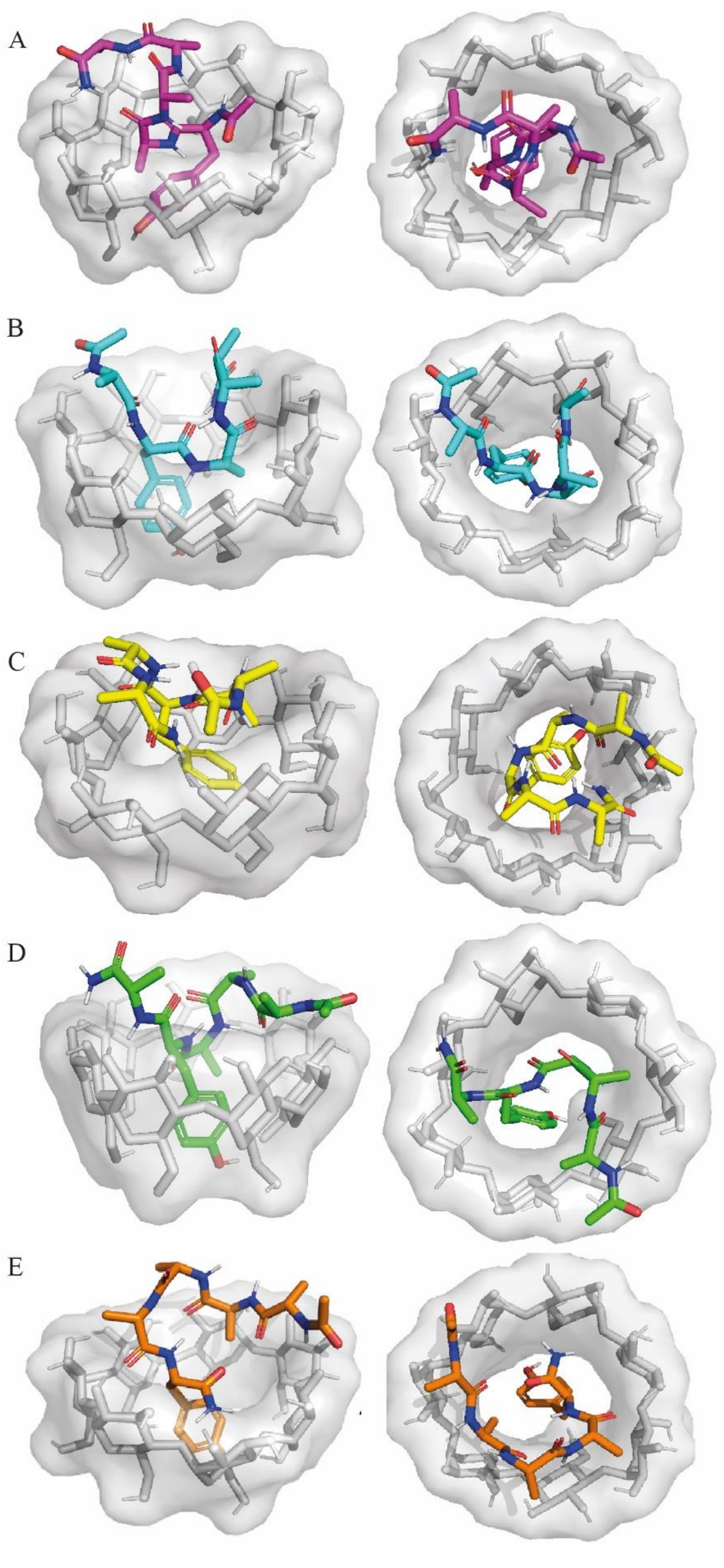

Figure S2. Molecular docking models for β-CD complexes with Tyr1(A), Tyr2 (B), Tyr3 (C), Tyr4 (D) and Tyr5 (E).

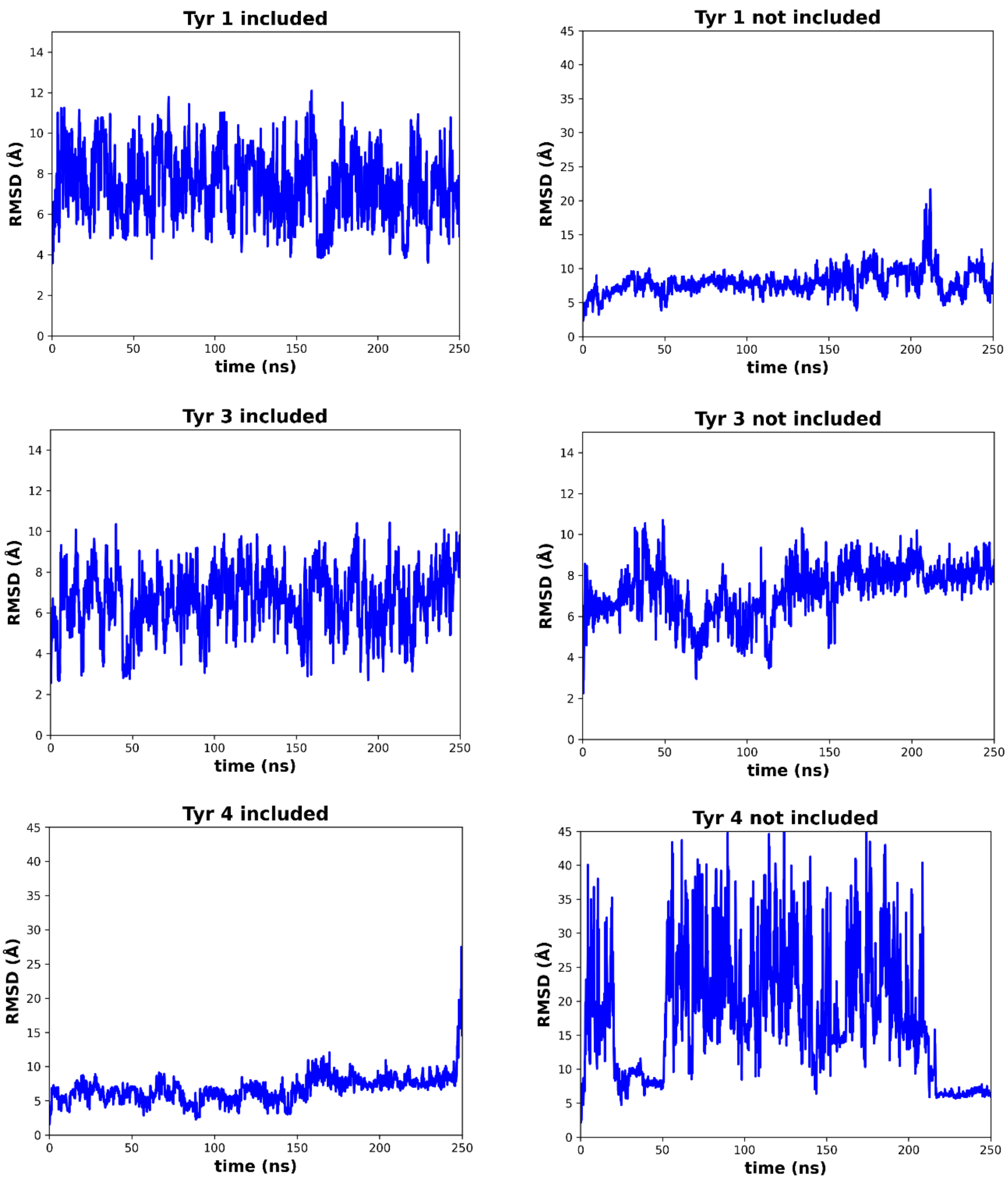

Figure S3. RMSD values calculated aligning the trajectories over the β-CD and tacking the respective docking poses as reference structures (R2)

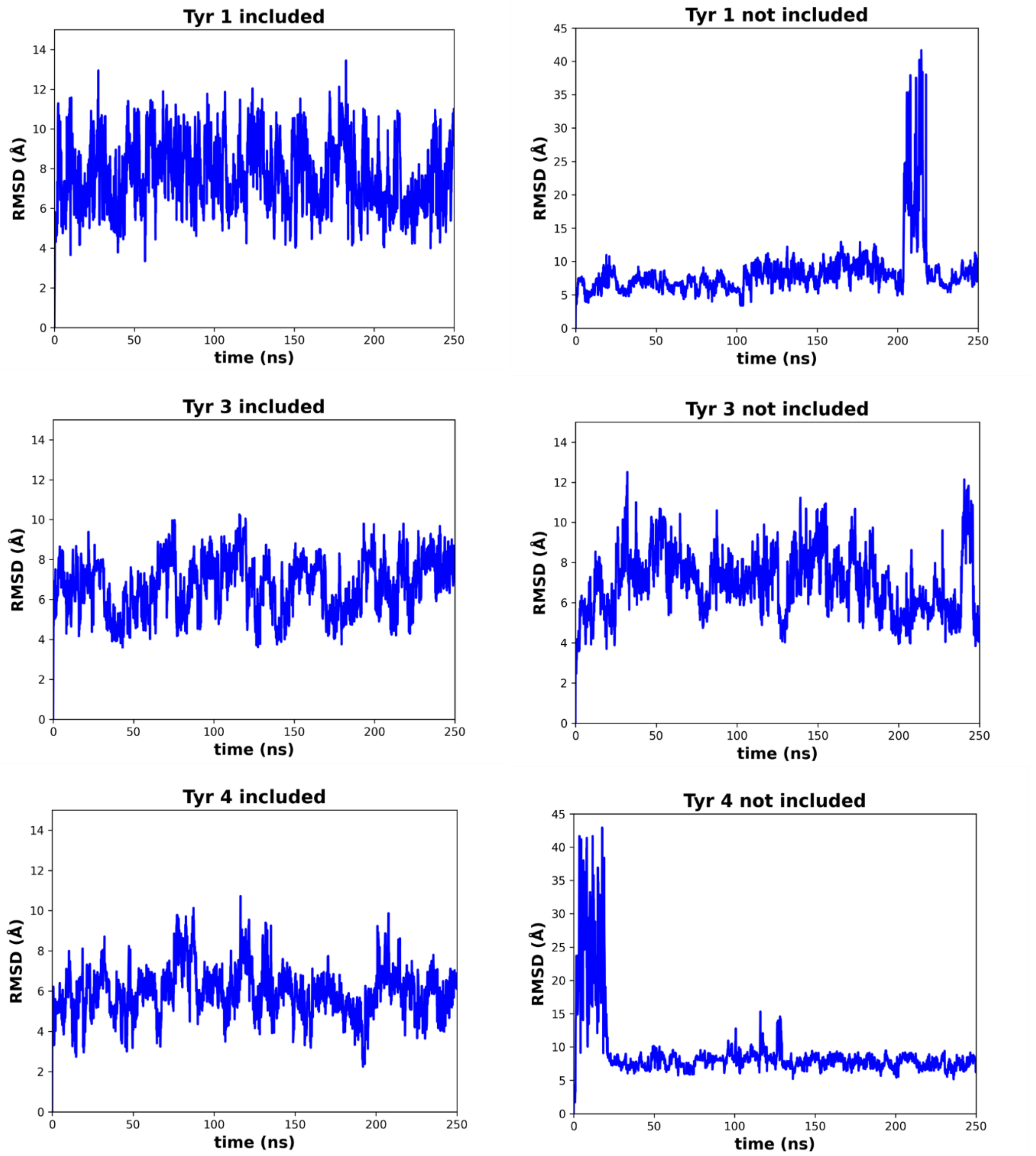

Figure S4. RMSD values calculated aligning the trajectories over the β-CD and tacking the respective docking poses as reference structures (R3)
